# Targeting of hyperactivated mTOR signaling in high-risk acute lymphoblastic leukemia in a pre-clinical model

**DOI:** 10.18632/oncotarget.2842

**Published:** 2014-12-02

**Authors:** Md. Nabiul Hasan, Manon Queudeville, Luca Trentin, Sarah Mirjam Eckhoff, Ilaria Bronzini, Chiara Palmi, Thomas Barth, Giovanni Cazzaniga, Geertruy te Kronnie, Klaus-Michael Debatin, Lüder Hinrich Meyer

**Affiliations:** ^1^ Department of Pediatrics and Adolescent Medicine, Ulm University Medical Center, Ulm, Germany; ^2^ Department of Women's and Children's Health, University of Padova, Padova, Italy; ^3^ Centro Ricerca Tettamanti, Clinica Pediatrica, University of Milano-Bicocca, Monza, Italy; ^4^ Institute for Pathology, Ulm University Medical Center, Ulm, Germany

**Keywords:** Acute lymphoblastic leukemia, pediatric, xenograft model, mTOR hyperactivation, preclinical targeting

## Abstract

Despite increasingly successful treatment of pediatric ALL, up to 20% of patients encounter relapse. By current biomarkers, the majority of relapse patients is initially not identified indicating the need for prognostic and therapeutic targets reflecting leukemia biology. We previously described that rapid engraftment of patient ALL cells transplanted onto NOD/SCID mice (short time to leukemia, TTL^short^) is indicative of early patient relapse. Gene expression profiling identified genes coding for molecules involved in mTOR signaling to be associated with TTL^short^/early relapse leukemia.

Here, we now functionally address mTOR signaling activity in primograft ALL samples and evaluate mTOR pathway inhibition as novel treatment strategy for high-risk ALL *ex vivo* and *in vivo*. By analysis of S6-phosphorylation downstream of mTOR, increased mTOR activation was found in TTL^short^/high-risk ALL, which was effectively abrogated by mTOR inhibitors resulting in decreased leukemia proliferation and growth. In a preclinical setting treating individual patient-derived ALL *in vivo*, mTOR inhibition alone, and even more pronounced together with conventional remission induction therapy, significantly delayed post-treatment leukemia reoccurrence in TTL^short^/high-risk ALL.

Thus, the TTL^short^ phenotype is functionally characterized by hyperactivated mTOR signaling and can effectively be targeted *ex vivo* and *in vivo* providing a novel therapeutic strategy for high-risk ALL.

## INTRODUCTION

Acute lymphoblastic leukemia (ALL) is the most frequently diagnosed malignancy among children and adolescents. Over the last decades, treatment has become increasingly successful leading to five-year event-free survival rates of more than 80%. This success is based on the use of risk adapted multi-agent chemotherapy protocols in which patients are assigned to therapy regimens with different intensities according to the individual probability to relapse [1]. Despite these major achievements in treatment of pediatric ALL, therapy fails in about 20% of patients resulting in relapse associated with inferior prognosis, especially if the disease reoccurs at early time points [2]. However, the majority of patients encountering relapse are not identified by currently established prognostic markers including detection of minimal residual disease (MRD), but are initially stratified into standard- or intermediate risk groups [3, 4]. These limitations clearly highlight the need for additional treatment modalities including improved risk stratification and novel therapies directed against identified targets.

Analysis of primary ALL cells is restricted since they are of limited availability and cannot be sufficiently cultured *in vitro*. Xenotransplantation of human leukemia cells onto immunodeficient mice leads to disease manifestation recapitulating the human leukemia in the recipient animals thereby allowing functional analysis of primary leukemia cells but also preclinical evaluation of novel therapies *in vivo* [5].

Previously, we investigated engraftment properties in a series of primary patient ALL samples transplanted onto NOD/SCID mice and described that a short time to leukemia manifestation in the recipient animals (time to leukemia short, TTL^short^) is associated with poor patient outcome and of strong impact for early relapse prognostication. Importantly, this engraftment phenotype is characterized by a specific gene expression profile including genes coding for regulators of cellular growth and proliferation. In particular, this signature shows low gene expression of molecules inhibiting mTOR and high transcript levels of mTOR activators suggesting increased mTOR signaling activity in this high-risk ALL subgroup [6].

In this study we now investigate the functional activity of this key survival pathway and evaluate mTOR as a molecular target for directed therapy in high-risk leukemia *ex vivo* and in a preclinical model setting *in vivo*.

## RESULTS

### The TTL signature is associated with mTOR signaling and inhibition

**Figure 1:**
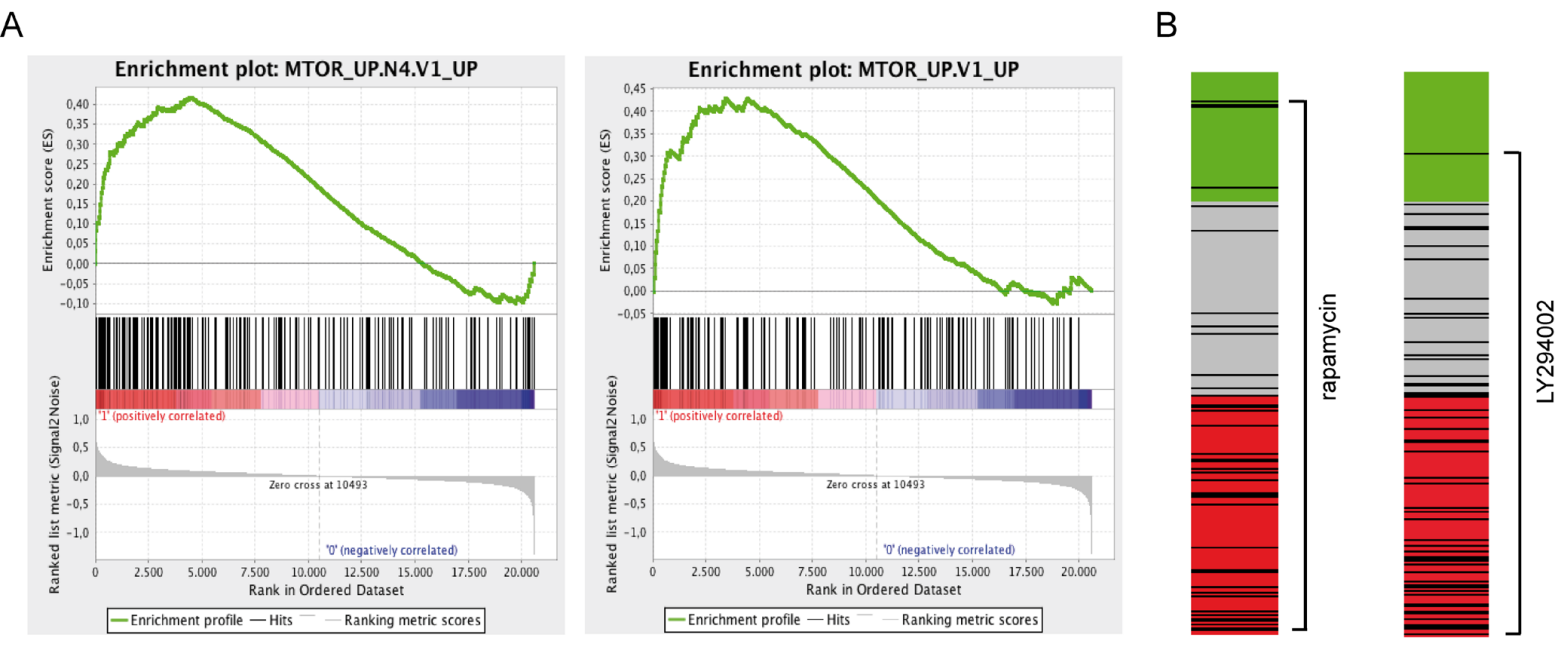
The TTL expression profile is associated with mTOR signaling and inhibition (A) Significant enrichment of two mTOR-annotated gene sets in the TTL^short^ profile (Gene Set Enrichment Analysis, FDR q-value ≤ .01). Enrichment plots depict enrichment scores (green lines) reflecting the appearance of members of the annotated gene sets (black vertical lines) along the gene list ranked from TTL^short^ (red) to TTL^long^ (blue). (B) Negative connection of the TTL^short^ set of genes with rapamycin and LY294002 induced gene sets (connectivity map analysis, cmap; mean connectivity score/specificity −0.37/0.29, and −0.44/0.32, respectively). Bar views depict drug treatment instances (black lines; rapamycin, n=44 and LY294002, n=61) of the cmap data set ordered by the respective connectivity score (green, positive; grey, null; red, negative).

We re-interrogated our previously obtained gene expression data set [6] and employed analysis tools assessing similarities of the TTL profile with gene sets annotated to different pathways or treatment-specific expression profiles (gene set enrichment and connectivity map analysis). By gene set enrichment analysis we investigated associations of the TTL profile with pathway-annotated gene sets (189 gene sets, Molecular Signature Database, C6: oncogenic signatures; http://www.broadinstitute.org/gsea/msigdb). Interestingly, a significant association (FDR q-value ≤ .01) was identified for two mTOR-annotated gene sets, highlighting the impact of mTOR signaling on NOD/SCID/huALL engraftment and patient outcome (FIGURE 1A, SUPPLEMENTARY TABLE ST1). In addition, we used a chemical genomics approach and analyzed similarities of the TTL signature with genome wide expression profiles obtained from human cancer cells lines treated with different bioactive molecules (connectivity map analysis). Most interestingly, the mTOR inhibitor rapamycin (sirolimus) and LY294002, an early synthetic PI3K inhibitor which is also active against mTOR, were identified with a negative connectivity score indicating their potential to revert the TTL^short^ phenotype (FIGURE 1B, SUPPLEMENTARY TABLE ST2).

Thus, these findings emphasize that mTOR signaling is involved in driving the TTL^short^ phenotype and that mTOR pathway inhibition by e.g. rapamycin is potentially effective to target high-risk ALL.

### Patient-derived ALL xenograft samples

Fourteen B-cell precursor (BCP) ALL xenograft samples (TTL^short^, n=7; TTL^long^, n=7) were investigated in this study. Short NOD/SCID/huALL engraftment was associated with inferior patient outcome in contrast to relapse-free survival of leukemias with a long engraftment phenotype also in this patient sample group, as previously observed (SUPPLEMENTARY FIGURE SF1). TTL phenotypes were not associated with prognostic leukemia characteristics and subgroups, all samples were *BCR*/*ABL* negative, one TTL^short^ leukemia carried a *MLL*/*AF4* and one TTL^long^ an *ETV6*/*RUNX1* gene fusion. Additionally, we investigated cytokine receptor-like factor 2 (*CRLF2*), a gene coding for the thymic stromal lymphopoetin receptor (TSLPR), which regulates proliferation of hematopoietic cells by signaling via JAK/STAT and PI3K/mTOR pathways [7]. Alterations in CRLF2, i.e. *P2RY8*/*CRLF2* or *IGH@*/*CRLF2* gene fusions, point mutations (*CRLF2*F232C) and Janus kinase 2 mutations (*JAK2*R683G) are frequently associated with *CRLF2* overexpression, an expression profile similar to Ph^+^-ALL (“Ph- or *BCR*/*ABL*-like”), and involve aberrant PI3K/mTOR signaling [8-13]. Importantly, all 14 samples analyzed in this study did not harbor *CRLF2* or *JAK2* gene alterations and did not show high *CRLF2* transcript or TSLPR protein expression. In addition, we investigated alterations of *IKZF1*, a gene coding for the lymphoid transcription factor IKAROS and identified two TTL^short^ samples with *IKFZ1* deletions (TABLE 1).

**Table 1:** Characteristics of patients and derived ALL xenografts

		total		TTL^short^		TTL^long^	
		N	%	N	%	N	%
Total		14	100	7	100	7	100
Gender	Female	6	43	2	29	4	57
	Male	8	57	5	71	3	43
Age at diagnosis	1-9 years	10	71	4	57	6	86
	0-1 and >9 years	4	29	3	43	1	14
Immunophenotype	pro-B ALL	1	7	1	14	0	-
	c-ALL	10	72	3	43	7	100
	pre-B ALL	3	21	3	43	0	-
Gene alterations	*ETV6/RUNX1*	1	7	0	-	1	14
	*MLL/AF4*	1	7	1	14	0	-
	*BCR/ABL*	0	-	0	-	0	-
	*P2RY8/CRLF2*	0	-	0	-	0	-
	*IGH@/CRLF2*	0	-	0	-	0	-
	*CRLF2*F232C	0	-	0	-	0	-
	*JAK2*R683G	0	-	0	-	0	-
	*IKZF1* deletion	2	14	2	29	0	-
*CRLF2*/TSLPR expression	*CRLF2* transcript high	0	-	0	-	0	-
	TSLPR positive (FACS)	0	-	0	-	0	-
Hyperleukocytosis	<50 (1000/μl)	7	50	4	57	3	43
	>50 (1000/μl)	7	50	3	43	4	57
Prednisone response day 8	Good	13	93	7	100	6	86
	Poor	1	7	0	-	1	14
Remission at day 33	Yes	14	100	7	100	7	100
MRD risk group	MRD non-high-risk	13	93	7	100	6	86
	MRD high-risk	0	-	0	-	0	-
	No MRD available	1	7	0	-	1	14
Final risk group	Non-high-risk	12	86	6	86	6	86
	High-risk	2	14	1	14	1	14

### PI3K/mTOR signaling in TTL^short^ and TTL^long^ ALL

**Figure 2:**
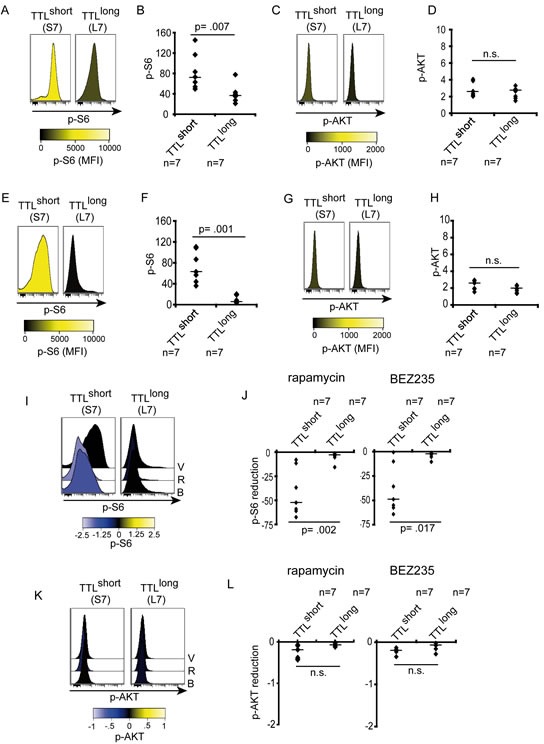
Increased downstream signaling activity in TTL^short^ ALL (A, B) High constitutive S6-phosphorylation (pS6 Ser235/236) in TTL^short^ but not TTL^long^ patient-derived ALL. (C, D) Similar low AKT-phosphorylation (pAKT Thr308) in all samples. (E, F) High S6-phosporylation maintained in TTL^short^ ALL upon culture in contrast to low pS6 in TTL^long^. (G, H) Low pAKT in both TTL subgroups. Histograms represent median fluorescence intensities (MFI), high (yellow) and low (black) phosphorylation according to the colorimetric scales. Diagrams show MFI relative to cellular autofluorescence of pS6 or pAKT, data points represent mean values of triplicate measurements for each sample, bars median values of TTL^short^ (n=7) and TTL^long^ (n=7) samples; Mann-Whitney U test; p, significance; n.s., not significant. (I, J) Rapamycin (R) and NVP-BEZ235 (B) reduce S6-phosporylation with equal effectivity in TTL^short^ but not TTL^long^ ALL. (K, L) No impact on pAKT by rapamycin and NVP-BEZ235 in both TTL subgroups. V, vehicle. Histograms represent median fluorescence intensities (MFI), arcsinh-transformed data, reduced (negative, blue) phosphorylation according to the colorimetric scales. Diagrams show reduction of MFI relative to cellular autofluorescene of pS6 or pAKT, data points represent mean reduction values of triplicate measurements for each sample, bars median reduction of TTL^short^ (n=7) or TTL^long^ (n=7) samples; Mann-Whitney U test; p, significance; n.s., not significant.

Based on our data pointing to critical involvement of mTOR signaling in TTL^short^/high-risk leukemia, we addressed mTOR pathway activity by investigating phosphorylation of key signaling molecules: ribosomal protein S6 (S6), a downstream molecule which is phosphorylated by the ribosomal protein S6 kinase (p70S6K1) upon mTOR activation; and AKT, an upstream signaling kinase that is activated by phosphatidylinositol 3-kinase (PI3K) also mediating mTOR activation. Phosphorylated S6 (pS6) and AKT (pAKT) were assessed by phosphoflow cytometry in primary leukemia cells isolated from ALL bearing recipients. Interestingly, different levels of constitutive pS6 were detected with higher S6 activation in TTL^short^ compared to lower activity in TTL^long^ leukemias (FIGURE 2 A, B). To validate our results obtained by flow cytometry, phosphorylation of signaling molecules was also assessed by western blot analysis showing significant stronger pS6 signals in TTL^short^ leukemias (SUPPLEMENTARY FIGURE SF2A, B). However, no differences in pAKT were observed in TTL subgroups as analyzed by flow cytometry (FIGURE 2C, D), concordant with no constitutive AKT phosphorylation (T308 and S473) analyzed by western blot in both TTL^short^ and TTL^long^ leukemia samples (SUPPLEMENTARY FIGURE SF2A). With this, the cytometry results are confirmed by western blot analysis validating this method for further analysis of pS6 and pAKT.

In addition to constitutive pathway activation, we assessed signaling after *ex vivo* culture in serum containing medium providing a general growth stimulus. Most interestingly, high S6 phosphorylation was maintained in TTL^short^ primografts in contrast to low pS6 in TTL^long^ leukemias upon culture (FIGURE 2E, F). Corresponding to similar low constitutive AKT activation, no differences in pAKT were detected after culture in all samples (FIGURE 2G, H).

Moreover, we also analyzed STAT5 phosphorylation and detected similar pSTAT5 levels with no significant differences between TTL^short^ and TTL^long^ leukemias (SUPPLEMENATRY FIGURE SF3).

Taken together, TTL^short^/high-risk leukemias are characterized by highly activated constitutive mTOR signaling maintained upon *ex vivo* culture, in contrast to low and decreasing mTOR activity in TTL^long^ ALL. Interestingly, no differential AKT activation was detected, suggesting that mTOR activation of TTL^short^ ALL is not regulated by upstream PI3K/AKT signaling.

### mTOR hyperactivity in TTL^short^ ALL is effectively inhibited *ex vivo*

Given our results of hyperactivated mTOR signaling in TTL^short^ leukemia, mTOR and PI3K signaling activities were addressed in response to pathway inhibition using the prototypic allosteric mTOR inhibitor rapamaycin and the dual mTOR/PI3K inhibitor NVP-BEZ235. Both inhibitors clearly reduced pS6 in TTL^short^ leukemias while pS6 levels in TTL^long^ remained unaffected. Interestingly, concurrent PI3K/mTOR inhibition (NVP-BEZ235) led to similar repression of pS6 as inhibition of mTOR by rapamycin alone (FIGURE 2I, J; SUPPLEMENTARY FIGURE 4). Rapamycin or NVP-BEZ235 did not affect pAKT in both TTL phenotype groups (FIGURE 2K, L; SUPPLEMENTARY FIGURE 4), corresponding to similar levels of low upstream PI3K/AKT signaling activity and no induction of a positive feedback mechanism.

Thus, our data indicate that hyperactivated mTOR signaling in TTL^short^/high-risk ALL can be effectively targeted. In line with low upstream AKT activity, mTOR inhibition alone was equally effective as dual PI3K/mTOR inhibition.

### Effects of mTOR pathway inhibition in high-risk ALL

**Figure 3:**
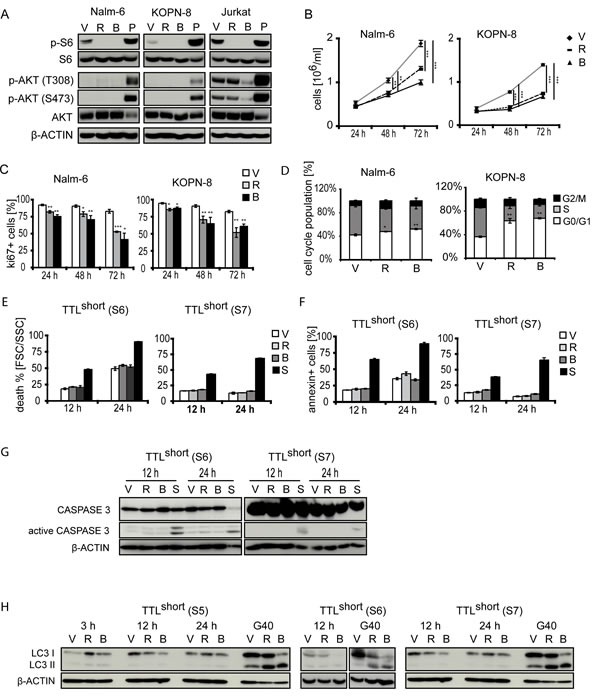
Effects of mTOR inhibition in BCP-ALL (A) Rapamycin (R) and NVP-BEZ235 (B) reduce pS6 but not pAKT in BCP-ALL cell lines Nalm-6 and KOPN-8. Note AKT-phosphorylation and reduction by NVP-BEZ235 in *PTEN* mutated T-ALL (Jurkat). V, vehicle; P, pervanadate incubated ALL cells (positive control). Upon rapamycin (R) and NVP-BEZ235 (B) exposure: (B) decreased cell growth, (C) decreased Ki67-positivity, and (D) smaller proportions of cells in S-phase in Nalm-6 and KOPN-8 cell lines. (E) No cell death induction, (F) absent Annexin-V positivity, (G) absent caspase 3 cleavage, and (H) absent conversion of LC3 in TTL^short^ primografts (S5, S6 and S7) (G40 glioblastoma cells, positive control) upon rapamycin and NVP-BEZ235 exposure after different time points. S, staurosporine inducing apoptosis (positive control). Data points and columns represent mean values of triplicate measurements with corresponding standard deviations; Student's T test compared to vehicle; p, significance, *< .05, **< .01, ***< .001.

Given our findings of hyperactivated mTOR signaling and susceptibility for pathway inhibition in TTL^short^ ALL, we next analyzed effects and mechanisms of mTOR inhibition in TTL^short^ leukemia. Primary ALL cells are not able to expand and proliferate if cultured *ex vivo* in contrast to cell lines showing *in vitro* growth. To address effects on cell proliferation *in vitro*, two BCP-ALL cell lines (Nalm-6, KOPN-8) were used. Consistent with our findings in primary ALL samples, rapamycin and NVP-BEZ235 reduced pS6 in both cell lines while pAKT remained unaffected (FIGURE 3A).

However, Jurkat T-ALL cells with a phosphatase and tensin homolog (*PTEN*) mutation showed constitutive AKT-phosphorylation on both residues T308 and S473 which was inhibited by the dual PI3K/mTOR inhibitor NVP-BEZ235, while no pAKT was detected in the BCP-ALL cell lines (FIGURE 3A). Proliferation and cell cycle progression were suppressed by both inhibitors to a similar extent indicated by decreased cell numbers upon culture, reduction of Ki67-positive cells, and lower proportions of cells in S-phase upon drug exposure (FIGURE 3B, C, D). In primary TTL^short^ ALL cells, both inhibitors showed no increase in cell death, Annexin-V positivity, caspase 3 cleavage or LC3 conversion at different time points, indicating no induction of overall cell death, apoptosis or autophagy (FIGURE 3E, F, G, H).

**Figure 4:**
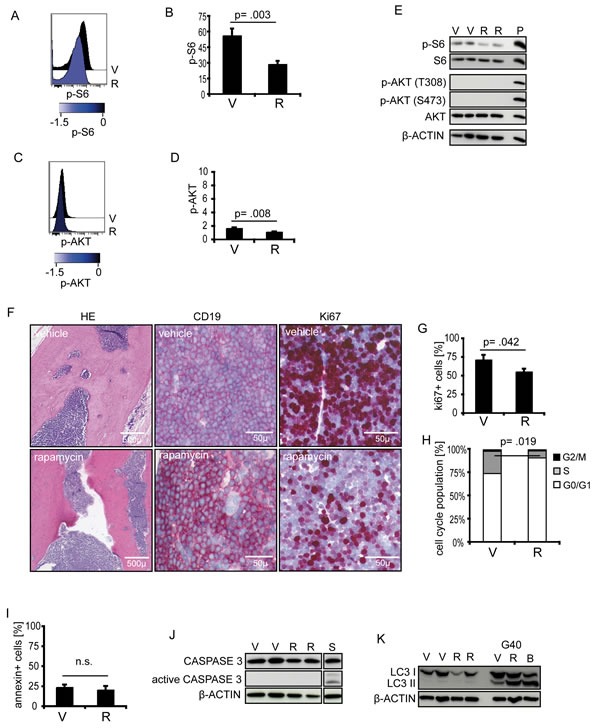
Effects of rapamycin on TTL^short^ ALL *in vivo* TTL^short^ leukemia (S7) bearing mice were treated with rapamycin (R) or vehicle (V). (A, B, E) *In vivo* rapamycin treatment reduces pS6 (pS6 Ser235/236), while (C, D, E) pAKT (pAKT Thr308) remains low. Phosposignaling analyses by cytometry (n=3/group) and western blot (n=2/group). P, pervanadate incubated ALL cells (positive control). Histograms represent median fluorescence intensities (MFI), arcsinh-transformed data, reduced (blue) phosphorylation according to the colorimetric scale. Diagrams show MFI relative to cellular autofluorescene of pS6 or pAKT. (F) Bone marrow sections of vehicle (upper) or rapamycin treated (lower row) ALL bearing recipients, hematoxylin/eosin (HE), huCD19, and Ki67 staining showing reduced Ki67-positivity upon *in vivo* rapamycin treatment. (G) Decreased Ki67-positivity in splenic cell suspensions, (H) decreased proportions of cells in S-phase, (I) absence of Annexin-V positivity, (J) no caspase 3 cleavage, and (K) no LC3 conversion in TTL^short^ ALL upon *in vivo* rapamycin treatment (G40 glioblastoma cells, positive control). S, staurosporine inducing apoptosis (positive control). Columns represent mean values of triplicate measurements with corresponding standard deviations, Student's T test; p, significance; n.s., not significant.

Both inhibitors showed anti-proliferative *in vitro* activity. However, dual PI3K/mTOR inhibition was not superior to mTOR inhibition alone, a finding that is in line with our observation of low upstream PI3K/AKT activity. Therefore, we focused our further analyses on mTOR inhibition by rapamycin and investigated the effects upon *in vivo* treatment. Recipients carrying a TTL^short^/high-risk leukemia (S7) were treated with rapamycin or vehicle for 5 days and sacrificed. A significant pS6 reduction was identified upon *in vivo* rapamycin treatment (FIGURE 4A, B, E). However, similar low levels of pAKT were found in both treatment groups (FIGURE 4C, D, E) indicating no feedback activation of PI3K/AKT signaling upon *in vivo* mTOR inhibition. Rapamycin treatment led to reduced proliferative activity of leukemia cells infiltrated into the recipient's bone marrow (FIGURE 4F) and spleen (FIGURE 4G, H). No Annexin-V positivity, caspase 3 cleavage or LC3 conversion was detected indicating neither induction of apoptotic nor autophagic cell death upon *in vivo* treatment (FIGURE 4I, J, K; SUPPLEMENTARY FIGURE 5), in line with our results on primary and cell line BCP-ALL.

Taken together, rapamycin inhibits highly activated mTOR signaling in TTL^short^/high-risk ALL resulting in blocked proliferation of leukemia cells upon *ex vivo* exposure and, most importantly, after *in vivo* treatment of mice with manifest leukemia.

### Preclinical *in vivo* evaluation of mTOR inhibition

**Figure 5:**
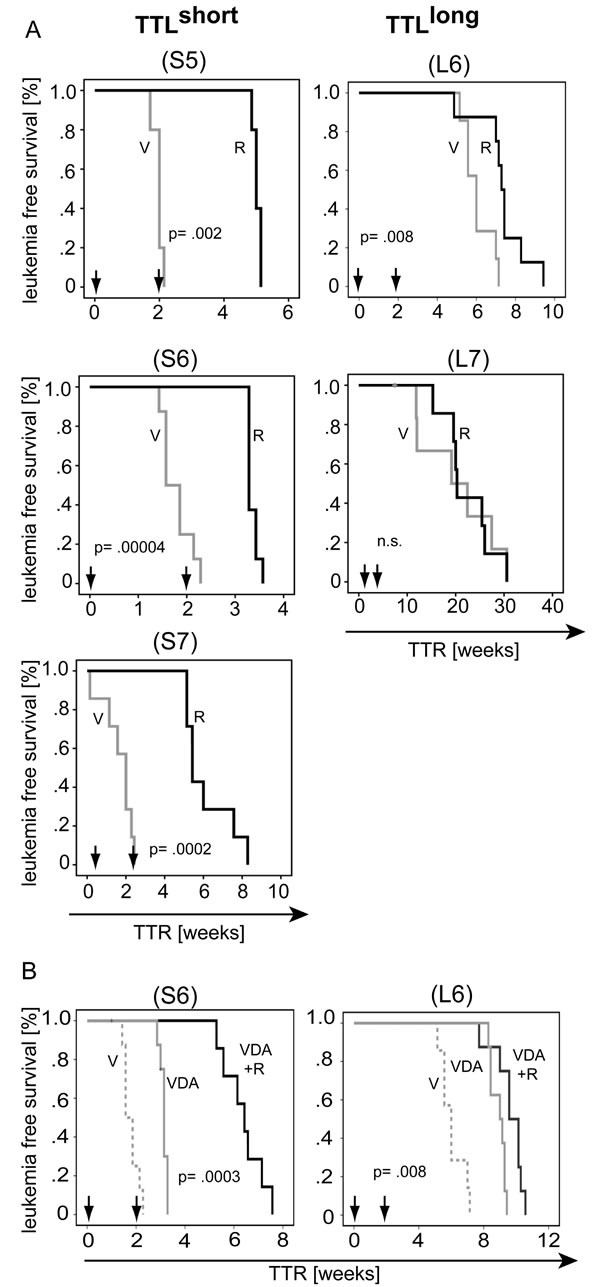
Preclinical targeting of mTOR hypercativation in TTL^short^ ALL (A) Rapamycin (R, solid dark line) leads to superior leukemia free survival compared to vehicle (V, solid grey line) treatment in hyperactivated mTOR/TTL^short^ primografts (S5, S6, S7), but not or to only a minimal delay in TTL^long^ ALL (L6, L7) bearing animals. (B) Superior survival of recipients bearing hyperactivated mTOR/TTL^short^ ALL (S6) upon remission induction chemotherapy and rapamycin (vincristine, dexamethasone, asparaginase and rapamycin; VDA+R, dark solid line) compared to chemotherapy alone (VDA, light solid line); in contrast to minimal prolonged survival in TTL^long^ leukemia (L6). (V, vehicle, dashed grey line). Arrows indicate 2 weeks treatment intervals; 5 to 8 animals per treatment group; TTR, time to leukemia reoccurrence; Kaplan Meier analysis, log rank test; p, significance; n.s., not significant.

Next, we evaluated mTOR inhibition as therapeutic approach to target hyperactivated mTOR/high risk/TTL^short^ ALL. Three prototypic TTL^short^ primografts showing characteristic high mTOR activity (S5, S6, S7) and two TTL^long^ leukemias (L6, L7) with low mTOR signaling were re-transplanted onto recipients. Upon ALL manifestation, vehicle (V) or rapamycin (R) was administered for a given time of two weeks and time until leukemia reoccurrence (time to reoccurrence, TTR) was quantified for each recipient. In all three TTL^short^ leukemias, a significant delay of post-treatment leukemia growth was observed upon rapamycin treatment (FIGURE 5A: S5, S6, S7). In contrast, rapamycin did not prolong leukemia free survival in TTL^long^ primografts or only to a small extent (FIGURE 5A: L6, L7).

In addition to single rapamycin treatment, we also evaluated rapamycin in combination with multi-agent remission induction chemotherapy used in pediatric protocols (vincristine, dexamethasone and asparaginase, VDA). Importantly, in TTL^short^/high-risk ALL combination of chemotherapy with rapamycin induced a clear superior survival compared to chemotherapy alone, in contrast to a minimal delay of few days in the TTL^long^ leukemia (FIGURE 5B; SUPPLEMENTARY TABLE ST3).

In summary, TTL^short^ ALL can successfully be treated by rapamycin showing preclinical effectivity of single treatment but more importantly also in combination with established chemotherapy elements, clearly emphasizing that inhibition of mTOR signaling is a potent therapeutic approach for patients suffering from high-risk ALL.

## DISCUSSION

Previously, we identified that *in vivo* proliferation of patient leukemia cells transplanted onto NOD/SCID mice is indicative of poor patient outcome and characterized by a specific transcript profile including genes coding for molecules regulating cell survival [6]. In this study, we now addressed signaling activity in leukemia cells on a functional level including preclinical *in vivo* evaluation of targeted pathway inhibition.

Re-investigating our gene expression data set employing gene set enrichment analysis, we identified enrichment of the TTL profile in mTOR-annotated gene sets further supporting that mTOR signaling is involved in driving rapid leukemia engraftment and early patient relapse. Moreover, using connectivity map analysis, we also found a significant overlap of the TTL profile with gene profiles induced by mTOR pathway inhibitors, again pointing to involvement of activated mTOR and to potential effectivity of its inhibition in TTL^short^ ALL. Intriguingly, on the functional level we found a clear difference in activity of mTOR signaling with hyperactivation in leukemia cells derived from TTL^short^/high-risk patients, which could be effectively targeted by rapamycin resulting in decreased growth, proliferation and successful *in vivo* treatment.

The serine/threonine kinase mTOR is a central downstream regulator shared by different pathways controlling cellular growth and survival [14]. Aberrant mTOR pathway activation has been reported in different cancers including hematologic malignancies by different mechanisms [15]. Mutational or functional loss of phosphatase and tensin homolog (PTEN), a molecule negatively modulating PI3K/AKT/mTOR signaling, results in resistance and promotion of T-ALL [16, 17]. In Philadelphia-chromosome positive (Ph^+^) B-lineage ALL, constitutive BCR/ABL tyrosine kinase activity directly drives PI3K/mTOR signaling and leukemogenesis [18-20]. Recently, a subtype of BCP-ALL has been described which, despite Ph-negativity, is characterized by a gene expression profile similar to the profile of Ph^+^-ALL and associated with poor outcome (“Ph- or *BCR*/*ABL*-like”) [21, 22]. Leukemias of this Ph-like subgroup frequently show gene alterations and dysregulated expression of cytokine receptor-like factor 2 (*CRLF2*) and activating Janus kinase (*JAK*) mutations [13]. Thymic stromal-derived lymphopoietin (TSLP), the ligand binding to CRLF2, induces leukemia proliferation involving mTOR signaling [7] and cases of Ph-like ALL with aberrant *CRLF2* expression displayed increased mTOR signaling activity [8, 23]. In contrast to these reports, none of the ALL samples included in our study carried *BCR*/*ABL* gene fusions, *JAK* mutations, *CRLF2* gene alterations or dysregulated *CRLF2*-transcript or surface receptor expression. Thus, mTOR hyperactivation in TTL^short^ BCP-ALL described in this study is not due to aberrant CRLF2, JAK or BCR/ABL activity. Alterations of the gene *IKZF1* are associated with inferior outcome and are frequently present in ALL with a Ph-like expression profile [22], however a direct association of *IKZF1* alterations and mTOR signaling has not been described. Two samples included into this study showed *IKZF1* deletions and exhibited a TTL^short^/poor prognosis phenotype, in line with the adverse outcome reported for patients with *IKZF1* alterations. Recently, genomic profiling of Ph-like ALL revealed different kinase-activating gene alterations associated with STAT5 activation [24]. Interestingly, we detected no different STAT5 signaling activities in leukemia samples of both TTL phenotypes suggesting that TTL^short^/high mTOR activity ALL is not associated with these gene alterations and corresponding upstream kinase activations.

No increased AKT-phosphorylation was observed in any of the patient-derived xenografts corresponding to absent PI3K/AKT signaling activity in our sample cohort. Similar to our findings, low or absent constitutive AKT activation was observed in *CRLF2* wildtype and a majority of Ph-negative BCP-ALL patient samples [8, 25]. Furthermore, specific *in vivo* AKT inhibition did not affect BCP-ALL growth in contrast to solid tumor xenografts [26], suggesting that leukemia survival is not controlled by the PI3K/AKT axis in these cases. Along this line, dual PI3K/mTOR inhibition in TTL^short^ ALL in our study was equally effective as mTOR inhibition alone, a finding which is consistent with low PI3K/AKT activity observed in our samples. Moreover, inhibition of mTOR, both *ex vivo* and *in vivo*, did not induce upstream AKT-phosphorylation, indicating that a positive feedback mechanism, leading to activation of upstream PI3K/AKT signaling after mTOR inhibition described for malignancies with constitutive PI3K/AKT signaling including acute myeloid leukemia [27, 28], is not operative in TTL^short^ ALL. Accordingly, we also observed absent PI3K/AKT signaling and no positive feedback loop in two BCP-ALL cell lines. Importantly, in contrast to our findings in BCP-ALL, active PI3K/AKT signaling was seen in the *PTEN* mutated Jurkat T-ALL cell line.

With this, we identified mTOR hyperactivation as potential driver for TTL^short^/high-risk ALL. However, unlike to other ALL subtypes as described above, increased mTOR signaling in TTL^short^ ALL is activated independently of upstream PI3K/AKT signaling. In our previous study characterizing TTL^short^ engraftment by expression profiling, genes coding for mTOR modulating molecules have been identified to be highly de-regulated in line with the molecules' functions inhibiting or activating mTOR (*DDIT4L*^low^/*RHEB*^high^/*FRAP1*^high^) [6]. Most importantly, the regulators identified interact directly with mTOR not involving signaling through PI3K/AKT: RHEB (Ras homolog enrichend in brain) binds specifically to mTOR complex 1 thereby leading to its activation [29], *FRAP1* codes for mTOR itself, and DDIT4L (DNA-damage-inducible-transcript-4-like) inhibits mTOR acting downstream of AKT [30], suggesting direct modulation of mTOR signaling independently of PI3K/AKT.

We showed that hyperactivated mTOR signaling can be efficiently repressed, identifying a therapeutic target in these high-risk leukemias. Given the central function of mTOR in regulation of cellular growth and proliferation, its inhibition is approached by different substances including rapamycin-derivatives and dual mTOR/PI3K inhibitors [31]. However, based on our results of inapparent PI3K/AKT signaling with no superior effectivity of dual PI3K/mTOR inhibition, we focused our further investigations on principle effects and the impact of mTOR inhibition including its pre-clinical effectivity in TTL^short^/high-risk ALL per se, using rapamycin as a prototypic mTOR inhibitor.

Inhibition of mTOR signaling resulted in decreased cellular proliferation *in vitro*, and importantly also in patient-derived leukemias upon *in vivo* therapy. In line with these findings in our study, repression of proliferation and cell growth by rapamycin or derivatives was reported for BCP-ALL cell lines and primary patient or xenograft samples [23, 32-35]. Despite this potent anti-proliferative effect of rapamycin on ALL, diverging findings on induction of apoptosis or autophagy by mTOR inhibition were described. Consistent with our results showing no apoptotic or autophagic cell death *ex vivo* and upon *in vivo* treatment, no apoptosis was induced upon exposure of primary (non-Ph^+^) BCP-ALL cells to rapamycin [36], or only in a subset of patient BCP-ALL samples while no apoptosis was detected in the remaining specimens [37]. Along this line, the rapalogue everolimus induced apoptosis only to a limited extend but induced autophagy in single BCP-ALL samples [33, 34]. However, growth delay of different xenografted B-lineage ALL samples sensitive to mTOR inhibition was observed demonstrating anti-proliferative *in vivo* activity [23, 33, 35, 38]. In line, we observed significantly postponed leukemia reoccurrence upon rapamycin treatment in animals engrafted with TTL^short^ leukemias indicating *in vivo* effectivity on mTOR driven TTL^short^/high-risk ALL. However, no such effect was observed in recipients carrying TTL^long^ ALL, pointing to mTOR-independent growth in this favorable subtype.

In a clinical situation, novel compounds will not be applied as single agents but along with established therapy regimens. Particularly, in order to efficiently incorporate mTOR inhibition into clinical treatment strategies, combination with cell death inducing drugs such as conventional chemotherapeutics will be required. We extended our preclinical analyses and included multi-agent chemotherapy resembling remission induction treatment and observed clearly increased activity of rapamycin together with chemotherapy in high-risk/TTL^short^ ALL. Similarly, synergism of mTOR inhibition and single cyotostatic drugs was reported in two B-lineage ALL xenografts (temsirolimus/methotrexate) and solid tumor samples (rapamycin/vincristine or cyclophosphamide) [39, 40]. In TTL^long^ phenotype ALL however, no clear superior survival upon combination treatment was observed.

Inhibition of mTOR is currently evaluated in relapsed/high-risk ALL in different clinical studies. However, TTL^short^/hyperactivated mTOR ALL characterized in this study is not associated with classical high-risk criteria [6], and signaling hyperactivity and susceptibility to mTOR inhibition might be heterogeneous among different individual leukemias. This clearly emphasizes the need for upfront identification of patients who would benefit from directed mTOR inhibition. Detection of aberrant signaling and pre-treatment evaluation of different inhibitors by cytometric phosphosignaling analysis of patient ALL specimens assessing individual pathway activity would be a feasible strategy and might be implemented into the routine diagnostic immunophenotypic work-up.

Taken together, we provide functional evidence that the TTL^short^/early relapse ALL is characterized by highly activated mTOR signaling driving *in vivo* leukemia growth. Hyperactivated mTOR signaling in TTL^short^ ALL was successfully targeted leading to significant superior leukemia free survival upon mTOR inhibition by rapamycin, and even more efficiently in combination with remission induction multi-agent chemotherapy pointing to potential benefit from combined mTOR inhibition and cytotoxic therapy for these high-risk patients. Most importantly, these poor prognosis patients are not defined by classical high-risk characteristics or recently described aberrations but could be identified by analysis of functional pathway activity.

## METHODS

### Ethics Statement

Investigation has been conducted in accordance with the ethical standards and according to the Declaration of Helsinki and according to national and international guidelines and has been approved by the author's institutional review board.

### Xenograft ALL

Xenograft ALL samples derived from BCP-ALL patients at diagnosis were established using our NOD/SCID/huALL xenotransplant mouse model and time to leukemia (TTL) was estimated as weeks from transplantation until disease onset as previously described [6, 41]. Seven TTL^short^ (S1-7) and 7 TTL^long^ (L1-7) leukemias (median TTL 8.6 and 22.0 weeks, respectively) were investigated. Patient samples were obtained after informed consent in accordance with the institution's ethical review board. Animal experiments were approved by the respective authority (Regierungspräsidium Tübingen, Versuch-Nr. 980). Patients were diagnosed and treated according to ALL-BFM 2000 or AIEOP-BFM ALL 2009 protocols (http://clinicaltrials.gov: NCT00430118; NCT01117441). Immunophenotyping was carried out following standard procedures [42]. *CRLF2* over-expression (20-fold above median expression in a cohort of 464 BCP-ALL cases) and *P2RY8-CRLF2* fusion were analyzed as previously described [43]. *IKZF1* deletions were investigated by Multiplex Ligation-dependent Probe Amplification (MLPA; SALSA MLPA P335-A3 ALL-IKZF1 probemix, MRC-Holland, Amsterdam, The Netherlands) according to the instructions of the manufacturer. Samples of pediatric ALL patients in complete remission were used as wild type controls. The fragments were separated on an ABI-3130 Genetic Analyzer instrument (Applied Biosystems, Monza, Italy) and data were analyzed using Coffalyser. Net software (MRC-Holland). Surface TSLPR was stained (allophycocyanin-conjugated anti-TSLPR; Biolegend, Fell, Germany) and assessed on a LSRII cytometer (BD, Heidelberg, Germany) with the corresponding isotype control. *IGH@CRLF2* gene fusions were analyzed by PCR (primer first exon *IGH*: 5′-AATACTTCCAGCACT-3′; primer third exon *CRLF2*: 5′-GTCCCATTCCTGATGGAGAA-3′; 94°C, 2 minutes; 30 cycles: 94°C, 30 seconds; 59°C, 30 seconds; 72°C, 1 minute; and 72°C, 10 minutes. *CRLF2* (F232C) and *JAK2* (R683G) mutations were analyzed on a GS-Junior-454 instrument (454 Life Sciences, Branford, CT, USA) as previously described [44]. Patient and xenograft characteristics are summarized in Table 1.

### Analysis of gene expression data

Based on our previously generated data set [6] (http://www.ncbi.nlm.nih.gov/geo; GSE13576), gene set enrichment analysis (GSEA, v2.0.14; http://www.broadinstitute.org/gsea) [45] was performed analyzing enrichment of a given gene set with 189 gene sets annotated in the Molecular Signature Database, C6: oncogenic signatures (http://www.broadinstitute.org/gsea/msigdb). The ‘gene_set’ permutation configuration was used, gene sets with NOM p-value ≤ .05 and FDR q-value ≤ .01 were considered significant. Connectivity map analysis (cmap build 02, http://www.broadinstitute.org/cmap) [46] was performed using the top differentially regulated probe sets (q-value ≤ .25; n=398) of our previously obtained TTL signature [6].

### *Ex vivo* analysis of leukemia cells

Xenograft leukemia cells were isolated from spleens of leukemia bearing mice transplanted with primary patient cells or already established xenografts and directly (without interim cryopreservation) subjected to analyses. Cell suspensions were prepared from infiltrated spleens and contained more than 90% of BCP-ALL cells as measured by flow cytometry. Cell lines (Nalm-6, KOPN-8 and Jurkat) were obtained from the Deutsche Sammlung von Mikroorganismen und Zellkulturen (Braunschweig, Germany) and stocks were frozen. Cells were thawed, passaged and authenticated by single tandem repeat profiling and subsequently used for experiments. Patient-derived cultured glioblastoma cells (G40) used as positive controls for mTOR inhibition induced LC3 conversion were kindly provided by Dr. A. Westhoff, Ulm. Cells were kept in RPMI 1640 medium supplemented with 10% fetal bovine serum and 1% L-glutamine (Gibco Life Technologies, Darmstadt, Germany) at 37°C in humidified air with 5% carbon dioxide. Cells were incubated with rapamycin or NVP-BEZ235 (LC Laboratories, Woburn, MA, USA) dissolved in dimethyl sulfoxide (DMSO; Sigma-Aldrich, Steinheim, Germany) or solvent. Primograft cells (L5) incubated with the phosphatase inhibitor pervanadate (20 μM, 3 hrs.) inducing maximum tyrosine-phosporylation were used as positive controls as previously described [8, 23].

S6-, AKT-, and STAT5- phosphorylation was assessed by phosphoflow cytometry as previously described [47] (Alexa-Fluor 647-conjugated anti-p-S6, Ser235/236; Alexa-Fluor 488-conjugated anti-p-AKT, Thr308; Cell Signaling, Beverly, MA, USA; and Alexa Fluor 647-conjugated anti-p-STAT5, Tyr 694, BD). For cell cycle analysis methanol-fixed cells incubated with RNase (Life Technologies, Darmstadt, Germany) were stained with 7-aminoactinomycin D (7-AAD; EMD Chemicals San Diego, CA, USA) [48]. Ki67 staining was carried out on paraformaldehyde-fixed (Polysciences, Eppelheim, Germany), saponin-permeabilized cells using Alexa-Fluor 488-conjugated anti-Ki67 (BD). Cell death was assessed by flow cytometry according to forward and side scatter criteria. Apoptotic cells were stained with Annexin-V-FLUOS (Roche, Mannheim, Germany) following the manufacturers instructions. Cells were assessed on a LSRII cytometer (BD) and data were analyzed using FlowJo 8.7 (Tree Star, Ashland, OR, USA) or Cytobank (http://www.cytobank.org) [49] software.

Western blot analysis and protein extraction were performed as previously described [41] using anti- p-S6 (S235/236), S6, p-AKT (T308), p-AKT (S473), caspase 3 (Cell Signaling, Beverly, MA, USA), LC3 (Thermo Scientific, Braunschweig, Germnay), AKT (BD), ß-ACTIN (Sigma-Aldrich), secondary horseradish-peroxidase-conjugated anti-rabbit or -mouse antibodies (Santa Cruz Biotechnology, Santa Cruz, CA, USA), and enhanced chemiluminescence (Thermo Scientific). Specificity of bands was identified by molecular weight markers. Densitometric quantification was performed using ImageJ 1.45s software [50].

Anti-Ki67 and -CD19 immunohistology staining was performed using the Dako Real Detection system based on alkaline phosphatase and permanent red chromogen for detection of bound antibody (Dako, CA, USA) as previously described [51]. Anti-active caspase 3 immunostaining (Abcam, Cambridge, UK) was performed as previously described [52].

### *In vivo* treatment

Upon appearance of 5% or more human ALL cells in peripheral blood, mice were randomized into groups and treated by intraperitoneal injections for 2 weeks with solvent (DMSO); rapamycin (2.5 mg/kg, 5 days/week); VDA (vincristine, 0.075 mg/kg, once/week; dexamethasone, 2.5 mg/kg, 5 days/week; asparaginase, 500 IU/kg, 5 days/week); or VDA and rapamycin. Mice were monitored and sacrificed at onset of leukemia related morbidity, confirming high leukemia infiltration in bone marrow, spleen and peripheral blood.

### Statistical analysis

Statistical analyses were carried out using the SPSS 19.0 software (IBM, Munich, Germany) applying the respective tests indicated, p-values lower than .05 were considered significant unless otherwise specified.

## SUPPLEMENTARY MATERIAL, TABLES AND FIGURES


