# Supplementary material for: Targeting of hyperactivated mTOR signaling in high-risk acute lymphoblastic leukemia in a pre-clinical model
Source: Oncotarget. 2014 Dec 2;6(3):1382–95. doi: 10.18632/oncotarget.2842 (PMC4359301; doi:10.18632/oncotarget.2842)
Supplement: Supplementary file 1 [file oncotarget-06-1382-s001.pdf]

Targeting of hyperactivated mTOR signaling in high-risk acute lymphoblastic leukemia in a pre-clinical model

Supplementary Material

Supplementary Table ST1: The TTL profile is significantly enriched in mTOR pathway annotated gene sets

Gene set enrichment analysis using our previously generated TTL gene expression data set. Twenty-one gene sets with a nominal p-value below .05 were identified including two mTOR pathway annotated data sets (Molecular Signatures Database, MSigDB; collection 6: oncogenic signatures). NES, normalized enrichment score; NOM p-val, nominal p-value; FDR q-val, false discovery rate q-value.

**gene set**	**NES**	**NOM p-val**	**FDR q-val**
IL2_UP.V1_UP	1,915	0,000	0,007
MTOR_UP.V1_UP	*1,792*	*0,000*	*0,010*
MTOR_UP.N4.V1_UP	*1,817*	*0,000*	*0,014*
LTE2_UP.V1_DN	1,693	0,000	0,020
CSR_LATE_UP.V1_UP	1,641	0,000	0,029
IL15_UP.V1_UP	1,592	0,002	0,040
STK33_NOMO_DN	1,440	0,007	0,136
BCAT_GDS748_UP	1,605	0,007	0,039
RPS14_DN.V1_UP	1,475	0,007	0,107
CSR_LATE_UP.V1_DN	1,483	0,008	0,114
STK33_SKM_UP	1,399	0,009	0,148
HOXA9_DN.V1_UP	1,405	0,011	0,163
RELA_DN.V1_UP	1,377	0,013	0,158
HOXA9_DN.V1_DN	1,403	0,016	0,154
STK33_UP	1,343	0,016	0,175
E2F1_UP.V1_DN	1,391	0,017	0,147
CRX_NRL_DN.V1_DN	1,371	0,029	0,149
P53_DN.V1_DN	1,330	0,034	0,188
BMI1_DN_MEL18_DN.V1_UP	1,358	0,036	0,160
SIRNA_EIF4GI_UP	1,372	0,038	0,155
RAF_UP.V1_DN	1,327	0,040	0,184

Supplementary Table ST2: The TTL signature is negatively connected with mTOR inhibitor induced gene sets

Connectivity map analysis was carried out employing our previously identified TTL expression profile (57 induced gene sets, p-value < .01). Within the top four gene sets significantly associated with the TTL profile (top three with negative connection), two gene sets induced by sirolimus (rapamycin) and the PI3K inhibitor LY-294002 were identified to be negatively connected indicating potential to revert the TTL^{short} phenotype.

rank	cmap name	mean connectivity score	number of instances	p-value	specificity
1	adiphenine	0,743	5	0,000	0,000
2	tanespimycin	-0,455	62	0,000	0,261
3	*LY-294002*	*-0,436*	*61*	*0,000*	*0,319*
4	*sirolimus*	*-0,366*	*44*	*0,000*	*0,295*
5	finasteride	0,568	6	0,000	0,014
6	Prestwick-692	0,703	4	0,000	0,012
7	etiocholanolone	0,459	6	0,000	0,000
8	pargyline	-0,736	4	0,001	0,000
9	PHA-00745360	0,380	8	0,001	0,027
10	podophyllotoxin	0,673	4	0,001	0,029
11	calcium folinate	0,668	5	0,001	0,017
12	vancomycin	0,674	4	0,001	0,000
13	biperiden	0,645	5	0,001	0,018
14	trifluoperazine	-0,485	16	0,002	0,164
15	metyrapone	-0,731	4	0,002	0,005
16	GW-8510	-0,670	4	0,002	0,176
17	fludrocortisone	0,323	8	0,002	0,065
18	alpha-estradiol	-0,451	16	0,002	0,093
19	trazodone	-0,718	3	0,002	0,023
20	promazine	-0,664	6	0,003	0,000
21	phthalylsulfathiazole	-0,669	5	0,003	0,035
22	meticrane	-0,692	5	0,003	0,015
23	colistin	0,668	4	0,003	0,006
24	CP-320650-01	0,292	8	0,003	0,041
25	sulfadimethoxine	0,550	5	0,004	0,040
26	2-aminobenzenesulfonamide	0,600	4	0,004	0,006
27	quinostatin	-0,817	2	0,004	0,092
28	8-azaguanine	-0,787	4	0,004	0,014
29	metrifonate	-0,627	5	0,004	0,005
30	apigenin	-0,714	4	0,004	0,038
31	ceforanide	0,664	4	0,004	0,028
32	famprofazone	-0,628	6	0,004	0,012
33	oxybuprocaine	-0,650	4	0,005	0,011
34	Prestwick-983	0,650	3	0,005	0,012

35	furazolidone	0,565	4	0,005	0,039
36	diloxanide	0,605	4	0,005	0,017
37	isometheptene	0,617	4	0,005	0,012
38	vinblastine	0,629	3	0,005	0,039
39	ginkgolide A	-0,692	4	0,005	0,020
40	nadolol	0,472	4	0,006	0,071
41	nortriptyline	-0,649	4	0,006	0,029
42	levonorgestrel	-0,561	6	0,006	0,066
43	isocarboxazid	0,463	5	0,006	0,006
44	tobramycin	-0,602	4	0,007	0,006
45	dapsone	0,498	5	0,007	0,011
46	lycorine	0,356	5	0,007	0,124
47	gentamicin	0,429	4	0,007	0,019
48	lisuride	0,362	5	0,007	0,084
49	practolol	-0,621	4	0,007	0,000
50	atractyloside	0,383	5	0,007	0,117
51	thioridazine	-0,395	20	0,007	0,323
52	ribavirin	0,429	4	0,008	0,019
53	karakoline	0,352	6	0,008	0,005
54	clomipramine	-0,647	4	0,009	0,061
55	chlorpromazine	-0,415	19	0,009	0,032
56	ethisterone	0,358	6	0,009	0,013
57	guanadrel	0,561	5	0,009	0,052

Supplementary Table ST3: Probabilities of leukemia free survival of TTL^{short} or TTL^{long} bearing recipients in response to *in vivo* treatment

leukemia	treatment	total	censored	events	median LFS [weeks]	SE	CI	p
**rapamycin**								
**S5**	vehicle	5	0	5	2.00	.09	1.83-2.16	
	rapamycin	5	0	5	5.00	.08	4.85-5.15	.002
**S6**	vehicle	8	0	8	1.57	.12	1.33-1.8	
	rapamycin	8	0	8	3.29	-	-	.00004
**S7**	vehicle	7	0	7	2.00	.26	1.49-2.50	
	rapamycin	7	0	7	5.43	.18	5.06-5.79	.0002
**L6**	vehicle	7	0	7	6.00	.26	5.49-6.57	
	rapamycin	8	0	8	7.28	.14	7.02-7.55	.008
**L7**	vehicle	7	1	6	19.14	6.38	6.62-31.66	
	rapamycin	7	0	7	20.28	.37	19.55-21.02	.9
**combination: rapamycin and remission induction chemotherapy**								
**S6**	VDA	8	1	7	3.14	.06	3.03-3.25	
	VDA+R	8	1	7	6.42	.37	5.69-7.16	.0003
**L6**	VDA	8	0	8	9.00	.50	8.01-9.99	
	VDA+R	8	0	8	9.57	.40	8.78-10.36	.008

LFS, leukemia free survival; SE, standard error; CI, confidence interval; p, significance (log rank test); and -, not applicable.

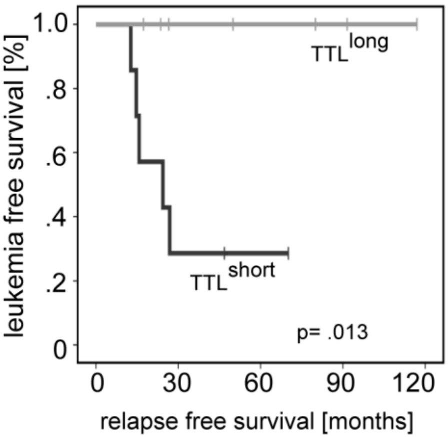

Supplementary Figure SF1: Superior relapse-free survival of patients with TTL^{long}

Patients whose ALL cells engraft with a long Time To Leukemia (TTL^{long}) phenotype show superior relapse-free survival in contrast to TTL^{short} patients in this sample cohort (N=14, TTL^{long} n=7, TTL^{short} n=7, Kaplan-Meier analysis, log rank test, p, significance).

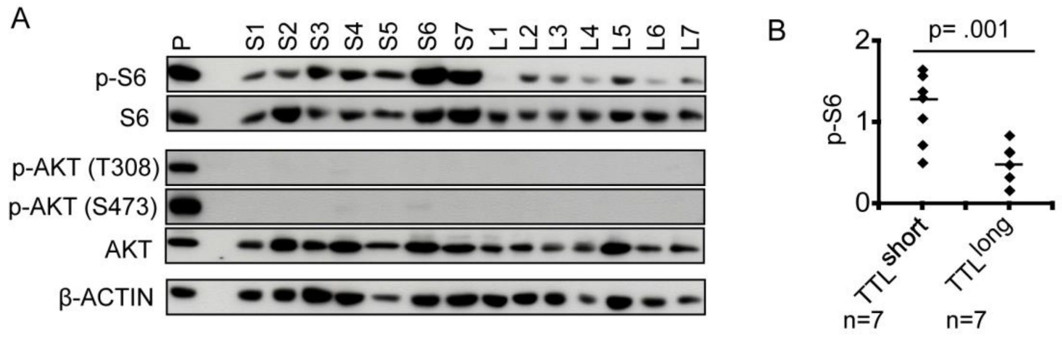

Supplementary Figure SF2: Western blot analysis of mTOR and PI3K signaling activity

Phospho-signaling analysis of pS6 and pAKT by western blot confirming the cytometric phosphoflow results. (**A**) High constitutive S6-phosphorylation in TTL^{short} (S1-7) compared to low pS6 signals in TTL^{long} (L1-7) patient-derived ALL xenograft samples. No AKT-phosphorylation in all samples. P, pervanadate incubated leukemia cells inducing maximum phosphorylation as positive control. (**B**) Significantly higher S6-phosphorylation in TTL^{short} ALL compared to TTL^{long} ALL (relative to total S6, quantified western blot signals, Mann-Whitney U test; p, significance).

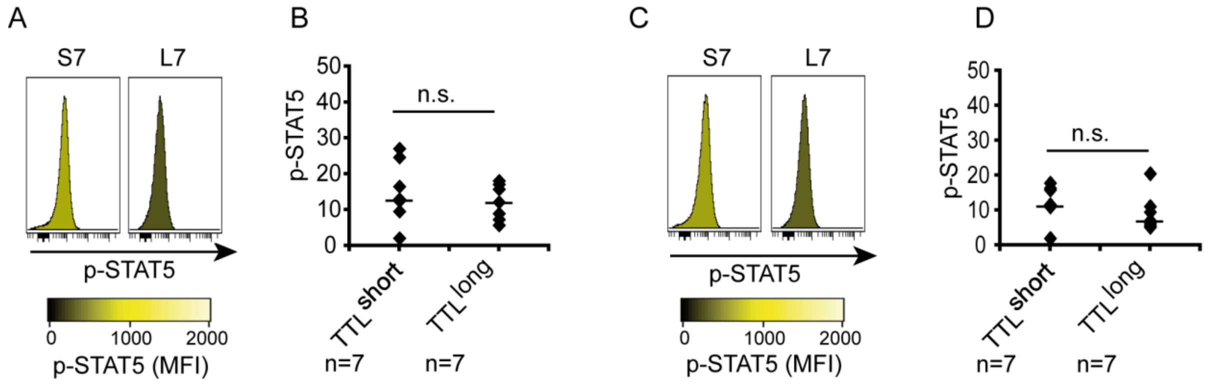

Supplementary Figure SF 3: Similar STAT5 activity in TTL^{short} and TTL^{long} ALL

Similar levels of STAT5 phosphorylation (pSTAT5 Tyr 694) in primograft ALL samples, (A, B) constitutively and (C, D) upon *ex vivo* culture. Histograms represent median fluorescence intensities (MFI), high (yellow) and low (black) phosphorylation according to the colorimetric scales. Diagrams show MFI relative to cellular autofluorescence of pSTAT5, data points represent mean values of triplicate measurements for each sample, bars median values of TTL^{short} (n=7) and TTL^{long} (n=7) samples; Mann-Whitney U test; n.s., not significant.

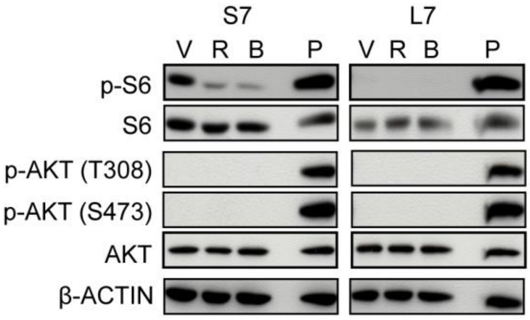

Supplementary Figure SF4: Inhibition of hyperactivated mTOR signaling in TTL^{short} ALL

Western blot phospho-signaling analysis of pS6 and pAKT upon exposure of two xenograft ALL samples with rapamycin (R) and NVP-BEZ235 (B) showing reduced S6-phosporylation in TTL^{short} (S7) but not TTL^{long} (L7) ALL while no effect on pAKT is observed in both TTL subgroups. V, vehicle. P, pervanadate incubated ALL cells (positive control).

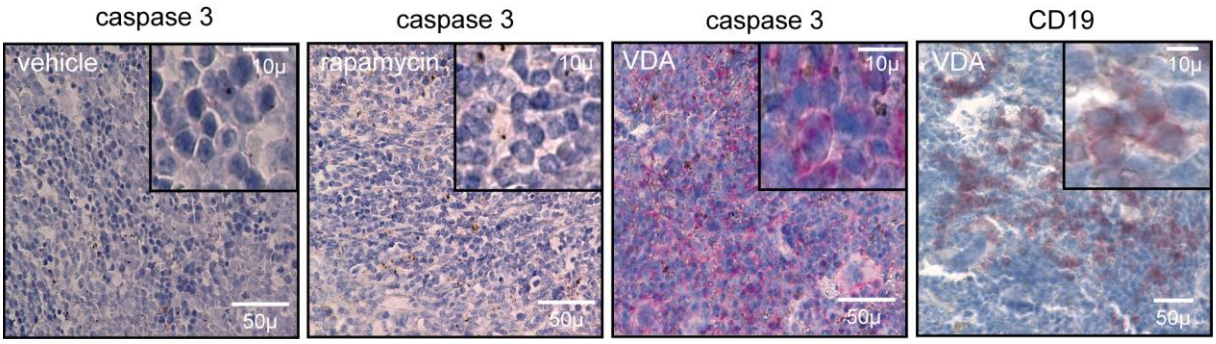

Supplementary Figure SF5: No apoptosis induction in TTL^{short} ALL upon rapamycin treatment *in vivo*

Spleen sections of ALL bearing recipients treated with vehicle, rapamycin (5 consecutive days), or remission induction chemotherapy (vincristine, dexamethasone asparaginase, VDA; one dose) stained for cleaved caspase 3 indicating apoptosis induction upon remission induction chemotherapy but not rapamycin treatment *in vivo*.
